# Leave or stay? Video-logger revealed foraging efficiency of humpback whales under temporal change in prey density

**DOI:** 10.1371/journal.pone.0211138

**Published:** 2019-02-05

**Authors:** Yu Akiyama, Tomonari Akamatsu, Marianne H. Rasmussen, Maria R. Iversen, Takashi Iwata, Yusuke Goto, Kagari Aoki, Katsufumi Sato

**Affiliations:** 1 Atmosphere and Ocean Research Institute, The University of Tokyo, Kashiwa, Chiba, Japan; 2 National Research Institute of Fisheries Science, Japan Fisheries Research and Education Agency, Kanazawa, Yokohama, Japan; 3 The University of Iceland’s Research Center in Húsavík, Húsavík, Iceland; 4 Sea Mammal Research Unit, School of Biology, University of St Andrews, Bute Building, St Andrews, Fife, United Kingdom; Pacific Northwest National Laboratory, UNITED STATES

## Abstract

Central place foraging theory (CPF) has been used to predict the optimal patch residence time for air-breathing marine predators in response to patch quality. Humpback whales (*Megaptera novaeangliae*) forage on densely aggregated prey, which may induce drastic change in prey density in a single feeding event. Thus, the decision whether to leave or stay after each feeding event in a single dive in response to this drastic change, should have a significant effect on prey exploitation efficiency. However, whether humpback whales show adaptive behavior in response to the diminishing prey density in a single dive has been technically difficult to test. Here, we studied the foraging behavior of humpback whales in response to change in prey density in a single dive and calculated the efficiency of each foraging dive using a model based on CPF approach. Using animal-borne accelerometers and video loggers attached to whales, foraging behavior and change in relative prey density in front of the whales were successfully quantified. Results showed diminishing rate of energy intake in consecutive feeding events, and humpback whales efficiently fed by bringing the rate of energy intake close to maximum in a single dive cycle. This video-based method also enabled us to detect the presence of other animals around the tagged whales, showing an interesting trend in behavioral changes where feeding duration was shorter when other animals were present. Our results have introduced a new potential to quantitatively investigate the effect of other animals on free-ranging top predators in the context of optimal foraging theory.

## Introduction

Predators should modify their foraging behavior to efficiently exploit prey whose density and availability dynamically changes over time. Since the study of optimal foraging began in 1966 [1, 2], various theories have been developed to predict the foraging decision of animals. These theories in general assumes that, as animal forage in a small-scale patch, the density of prey in the patch decreases over time, thus the rate of energy intake diminishes (diminishing return). Under this assumption, the optimal forging theory predicts the timing when the animal should stop feeding and leave the patch to maximize the energy intake (or certain currency) per unit time with minimum cost [3–5]. While many empirical tests in laboratories or carefully controlled field experiments have been performed supporting these theories [6], such studies were mostly restricted to terrestrial or captive animals that move in a relatively small area where visual observation can be conducted.

Great progress has been made over the last couple of years, with respect to the development of methods using electronic tags, that has enabled us to observe the behavior of predators and detect prey capture events using animal-borne accelerometer data, together with a camera or a video logger [7, 8], or an accelerometer data attached to the jaw or the head of a predator [9]. These developments have introduced a new era in the field of animal foraging studies, by enabling detailed tests for predictions of foraging theories in various free-ranging animals, especially air-breathing marine predators that are nearly impossible to directly observe [10–14].

Air-breathing marine predators, such as seabirds, marine turtles, and marine mammals, are considered “central place foragers,” [15] due to their need to surface (central place) between foraging dives to breathe air [4, 14]. The central place foraging theory (CPF) is the most effective model developed to predict how a predator maximizes the energy intake per unit time in relation to change in prey density under constraints: (1) the time cost of moving back and forth between patches and water surface; and (2) the post-surface time where they restore oxygen, because of the trade-off between energy intake and oxygen depletion associated with dives [4, 12, 14, 15]. Longer dives consume more oxygen, resulting in a longer post-surface recovery time [16, 17].

Among the diving predators, rorqual whales (*Balaenopteridae*) are extraordinary: they are the largest predators on earth and their magnificent body size has led to unique characteristics in the contexts of foraging ecology. While most predators target and capture a single prey at a time, rorquals forage on densely aggregated krill and schooled fish using a strategy known as lunge feeding [18, 19]. During a lunge, rorquals accelerate at a high speed to a patch of prey, engulf a vast amount of prey-laden water, and filter the prey from the water using baleen plates [20, 21]. Hence, a single engulfment during lunge may induce a drastic decrease in prey density in a single dive (patch), and the decision whether to leave or stay at the prey patch in response to this drastic change should have a significant effect on prey exploitation efficiency. However, whether rorqual whales show adaptive behavior in response to the diminishing prey density in a single dive has been technically difficult to test.

Foraging behavior of rorqual whales was previously studied by attaching multi-sensor digital archival tags onto whales, in combination with prey distribution data measured using ship-mounted echo-sounders [12, 22]. This method succeeded in providing many fruitful insights on the whales’ foraging strategy across patches, but the spatiotemporal resolution was not high enough to detect the change in prey density on a single-dive scale. In this study, we estimated the relative prey density around humpback whales (*Megaptera novaeangliae*) at high temporal resolution using a video logger attached to the whales. Using this information, we calculated the cumulative relative energy intake per each lunge and tested a hypothesis that humpback whales will adjust their foraging duration to maximize the rate of energy intake over a single-dive cycle.

**Fig 1:**
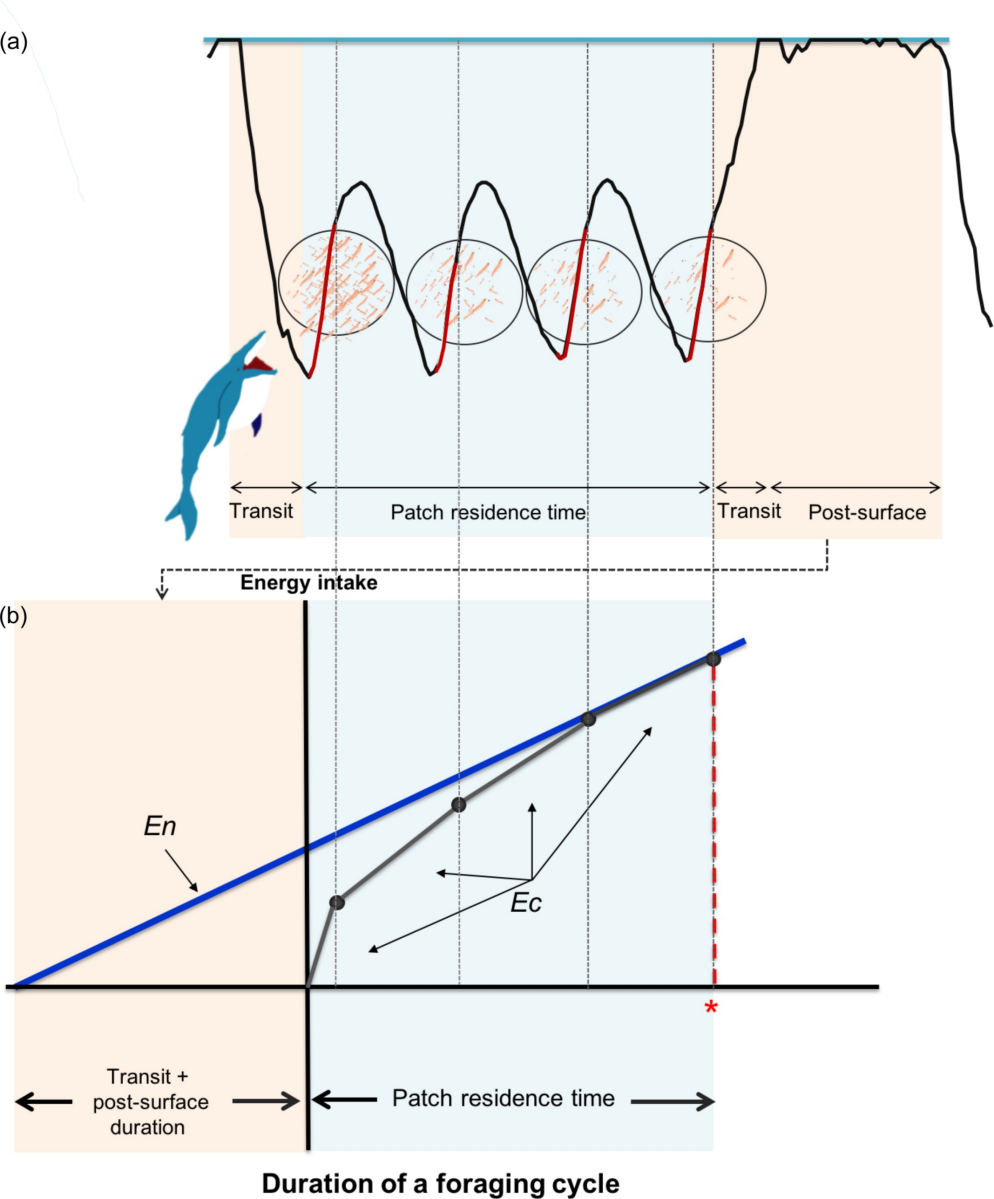
Illustration and diagram showing a gain function of a single dive with the optimal patch time according to the assumption of CPF. (a) A depth profile of a whale lunging (red lines) four times in a single dive. The energy intake of each lunge is the sum of the Index of Prey Density measured over the lunge duration. (b) Rate of energy intake is plotted as linear functions (*Ec*; black line on the right-hand side), with each black dot representing the duration and cumulative energy intake at each lunge. Here, the prey density is predicted to decrease after each lunge showing a shape of a diminishing rate of energy gain (slope of a line). The left-hand side shaded in pink indicates the transit duration (descent + ascent) and the post-surface duration. The blue line starting from the sum of the transit and surface duration to the last lunge point on the right-hand side indicates the total rate of energy intake (*En*) of this whole foraging cycle. The optimal patch time is indicated in red *, which in this case is where *Ec* and *En* overlaps.

We present a simple model to test the efficiency of each dive based on the CPF approach. Humpback whales in our study site (Skjálfandi Bay in northern Iceland) are mainly feeding on krill. Krill in North Atlantic Ocean are heterogeneously distributed in patches, highly variable in space and time [23]. In this study, foraging humpback whales are assumed to encounter a single patch of prey per dive. Foraging cycle of humpback whales in a single dive include, 1) a transit/dive phase to the underwater prey patch, 2) patch residence time, where actual lunge feeding phases with acceleration to high speed, engulfment of water and prey, and the filtration of water [19, 24] occur once to several times, and finally 3) transit to the surface, and post-surface phase where they restore oxygen (Fig 1A), a factor essential for a model of air-breathing divers [25]. In our study, we defined the energy gain per unit time over a single dive as a currency optimized by humpback whales and the travel time, post-surface recovery time, and change in rate of energy intake in relation to change in prey density over time as constraints. Under this condition, we hypothesize that, 1) the gain function within a single foraging dive will show a diminishing rate of energy intake (diminishing return) and 2) humpback whales will leave the prey patch when the total rate of energy intake (*En*) in a single dive cycle is maximized: *En* in a single dive cycle is maximized when it overlaps with the rate of energy intake (*Ec*) (Fig 1B).

Unexpectedly, this video-based method enabled us to detect the presence of other whales that may potentially affect the foraging behavior of the whale. We, therefore; also investigated how humpback whales adjust their foraging duration in response to presence/absence of other individuals.

## Materials and methods

### Field study and equipment

Tagging was conducted in Skjálfandi Bay off Húsavík in northern Iceland (66°05'N, 17°19'W), which is known as a feeding ground for many cetaceans [26, 27], from 31 May to 10 June in 2013, and 21 June to 30 June in 2014. Humpback whales were approached slowly and tags were deployed from a Zodiac inflatable boat (60-hp engine, 5-m long), using an 8-m carbon fiber pole with a tag set at the tip of the pole. Another boat was standing by for safety and to obtain photo-identification records of the whales. The tag was attached to the whale with a suction cup, which naturally detached after a few hours, and was retrieved using its VHF signal.

The animal-borne tag consisted of (1) an accelerometer sensor, W1000-3MPD3GT (26 mm in diameter, 175 mm in length, 140 g in air; Little Leonardo Corp., Tokyo, Japan), programmed to record tri-axial acceleration at 32 Hz; speed, depth, temperature, and tri-axial magnetometer sensors sampling at 1 Hz; (2) a video logger, DVL 400 (23 mm in diameter, 114 mm in length, 80 g in air, for 5 h of recording, 80° field-of-view on land; Little Leonardo Corp.), used in 2013, or DVL 400L (23 mm in diameter, 145 mm in length, 115 g in air, for 10 hours of recording, 80° field-of-view on land; Little Leonardo Corp.), used in 2014; (3) a suction cup (85 mm in diameter; Canadian Tire Corporation, Toronto, Canada); and (4) a VHF radio transmitter (10 mm in height, 10 mm in width, 55 mm in length, 22 g in air; Advanced Telemetry Systems, Isanti, MN, USA), all assembled in one float (NiGK Corporation, Tokyo, Japan).

### Body angle alignment, stroke, and pitch angle calculation from acceleration

The signals of tri-axial acceleration and tri-axial magnetism were adjusted to align the tag frame with respect to the body’s frame using MATLAB R2013a Student Version, per Johnson and Tyack [28]. These data were then used to obtain swimming stroke and pitch angle using IGOR Pro (WaveMetrics, Lake Oswego, OR, USA) following the method of Tanaka et al. [29] and Sato et al. [30]. The tri-axil acceleration data were separated to low-frequency gravity-based acceleration using the 0.1 Hz low-pass filter in the IFDL software in IGOR Pro. By subtracting this gravity-based acceleration from the original, high-frequency acceleration was obtained, reflecting the propulsive (stroking) activity. Among the tri-axial acceleration data, dorso-ventral axis was used for analysis. The value of low-pass filtering was determined as 0.1 Hz from visual observations of the data, and value from previous reports of humpback whales [17, 31, 32] as a reference. Finally, IGOR Pro (binomial smoothing, 30 passes) was used to smooth the high-frequency acceleration data and remove noise at frequencies above the stroke rate that is likely to represent vibration of the suction cup and the tag. The pitch angle of the whales was calculated from the low-frequency gravity-based acceleration and the tri-axial magnetism using ‘ThreeD_path’ macro compliant with IGOR Pro [33,34].

### Speed calibration

The swimming speed of an animal was calculated from the rotation counts of the propeller mounted on the accelerometer. Rotation counts were converted to speed with an equation obtained in a calibration experiment using an experimentally designed Blazka-type swim tunnel [35] with all five accelerometers (W1000-3MPD3GT). The accelerometers were set inside the tunnel and rotation counts were obtained under flow speeds ranging from 0.1 to 1.1 m s^-1^, and the results were plotted as a regression line. All five accelerometers yielded high correlation coefficients (Range: 0.991 to 0.999; n = 10). Stall speed was also determined from the experiment to be 0.2 m s^-1^ for all loggers. Speeds below this value were considered indistinguishable from zero [29]. The speed of an animal obtained from this calibration equation might not reflect the exact swim speed of the whale but is sufficient to observe the change in speed of discrete phases associated with lunge events [24] for this study.

### Dive and lunge events

The body diameter of humpback whales is reported to be 3.21 m [36], so the start and end of each dive was defined as when the whale descended below and ascended above 4 m in depth, and it was extracted using the package Ethographer 2.00 in IGOR Pro [37]. Lunge events are characterized by a rapid acceleration in speed and energetic stroking [31, 32]. Using these characteristics, previous studies have identified lunge events from swimming speed obtained by flow noise [18] or minimum specific acceleration and jerk [32]. To explore the definition of lunges, we detected lunge events from video data and visually inspected changes in acceleration and speed during lunges. Interestingly, even though there were variations in lunge speed, stroking effort was similar in all lunge events; therefore, the acceleration signal was used to extract the lunge events. A new method was used instead of jerk, because it was simpler and could systematically detect the feeding event just as well. Following the method of Sakamoto et al. [37], high-frequency strokes were identified by first generating a spectrum from acceleration signals of dorso-ventral axis. Then, each second of this spectrum was separated into four clusters by an unsupervised classification algorism, the *k*-mean clustering, and the two high-frequency clusters were considered the area of the lunge event. In some cases, however, high-frequency stroking was observed during the descent phase or at the surface. From visual observations in the field, as well as from the attached video logger, the whales would first dive underwater and accelerate from below toward the surface, but no lunge event started from the surface or during the descent phase; therefore, high frequency stroking detected at depths shallower than 4 m or at negative pitch angles signifying the descent phase, was removed from the lunge counts. The accuracy of this method was verified using video data of two whales (WhB13 and WhB14), which showed 96.8% detection match of lunge events (331 dives with 129 lunge events during 10.2 hours of video data; two unmatched and two visually indistinguishable lunge events from video). A single lunge duration was defined as the beginning to the end of the high-frequency stroking phase plus five seconds (Fig 1A, red line), which was the approximate duration of the deceleration phase after the peak speed where mouth closing occurs [24, 32]. Patch residence time was defined as the beginning of the first lunge of a dive extending to the end of the last lunge of the same dive (Fig 1A, light blue area).

### Video analysis

Video recordings were visually examined with VLC Media Player 2.0.2, and were synchronized with the accelerometer data from the surfacing phase of depth profile every 30 min.

**Fig 2:**
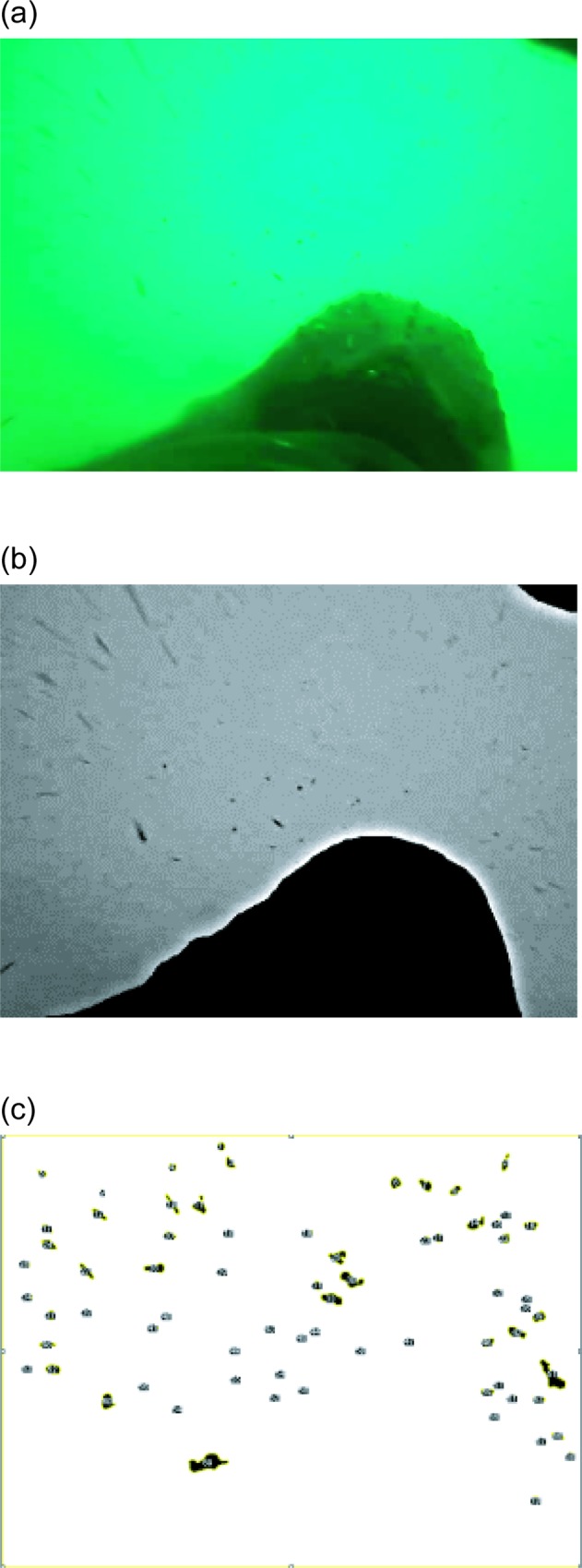
Example of video analysis for counting prey. (a) Video data converted to still image (b) Images converted to grey scale and filtered (3) Image with the body of the whale and large objects removed and the remaining objects (prey) being marked.

In order to calculate the relative energy intake of the whale while feeding, we first estimated the relative prey density in front of the whale. All videos recorded at 30 frames per second, were converted to still images of 640 × 480 pixels using Free Studio 6.4 (DVDVideoSoft Ltd., UK: Fig 2A). At the same time pictures were sampled every one second in order to avoid counting the same krill multiple times. These converted images were then imported to ImageJ software [38], within which all image analyses were conducted. Images were first converted to grey scale (8-bit) and filtered using the unsharp mask (radius 9.0, mask weight 0.7) and Gaussian blur (sigma 7) to make prey-like objects stand out from the background (Fig 2B). The body of the whale and any other large objects, such as dolphins or other whales, were cleared out manually from all images and the remaining objects (prey) within the frame were marked using the command *Find maxima* (Fig 2C). Finally, the number of objects and their area, the total area of the image, and the total area without large objects were calculated (in pixels) using the command *Analyze particle*. Using the values obtained, relative prey density, defined as the index of prey density (IPD) was first calculated every second by dividing the number of prey by the area without the body or any other large objects:

$$\mathrm{Index}\;\mathrm{of}\;\mathrm{prey}\;\mathrm{density}\;(\mathrm{IPD})=\frac{\mathrm{Number}\;\mathrm{of}\;\mathrm{prey}}{\mathrm{Area}\;(\mathrm{in}\;\mathrm{pixels})\;\mathrm{without}\;\mathrm{large}\;\mathrm{objects}}\;[\mathrm{unit}\cdot {\mathrm{pixels}}^{-1}].$$ (1)

When the whales were at the surface or close to the surface, water bubbles at the surface were mistakenly detected as prey, generating unrealistically high values; therefore, measurements obtained from depths shallower than 4 m (same as the defined dive depth) were omitted.

Beside the prey, video logger was carefully observed to find any other factors that may influence the whales’ behavior, such as encounter with other animals that could be their competitor or predator. Dives were categorized as “present” or “absent” when other animals were seen or not seen, respectively, in the underwater footage. Animals observed at the surface, and the problem of animals being in a blind spot, due to the narrow view angle of the video, were disregarded in this study to avoid arbitrary judgments.

### Foraging model

#### Rate of energy intake (*Ec*)

A gain function was constructed by calculating the energy intake of each lunge from IPD, where each lunge event is indicated by a red line in Fig 1A. Here, we assumed that density of prey (prey density in each frame) passing by the whales is proportional to the energy intake of filter-feeding animals, therefore; the sum of IPD during each lunge event was regarded as the relative energy intake of each lunge event. Cumulative of this relative energy intake (unit·pixels^-1^; termed cumulative energy intake, hereafter) during each lunge was plotted as a linear function (black line on the right-hand side of Fig 1B), with each point corresponding to the time and the cumulative energy intake during each lunge in that particular foraging dive. The slope inclination from the beginning of patch residence time to the end of the first lunge corresponded to the rate of energy intake of the first lunge (*Ec*: unit·pixels^-1^·sec^-1^). From the second lunges, the slope inclination between two lunges corresponded to the rate of energy intake (*Ec*: unit·pixels^-1^·sec^-1^) during the period from end of preceding lunge until the end of the subsequent lunge. This gain function was constructed for all dives with greater than or equaled to two lunges per dive.

#### Total rate of energy intake (*En*)

The total rate of energy intake per unit time during a single dive cycle (*En*) was calculated from the cumulative energy intake over the duration of each foraging cycle. The left-hand side of the horizontal axis of Fig 1B indicates the transit duration (descent + ascent) and the post-surface duration, which are the areas indicated in pink in Fig 1A. The slope of the blue line in Fig 1B, starting from the sum of the transit duration and the post-surface duration to the lunge point on the right-hand side, indicates the total rate of energy intake (*En*) during the last lunge:

$$\mathrm{Total}\;\mathrm{rate}\;\mathrm{of}\;\mathrm{energy}\;\mathrm{intake}\;(En)=\frac{\mathrm{Sum}\;\mathrm{of}\;\mathrm{cumulative}\;\mathrm{energy}\;\mathrm{intake}\;\mathrm{of}\;\mathrm{lunges}}{\mathrm{Duration}\;(\mathrm{Transit}+\mathrm{Post}‑\mathrm{surface}+\mathrm{Patch})}\;[\mathrm{unit}\cdot {\mathrm{pixels}}^{‑1}\cdot \sec.^{‑1}]$$ (2)

This total rate of energy intake (*En*) was calculated for all lunge points in a dive.

#### Investigating the efficiency

The concept of the CPF was applied to interpret the time efficiency of a foraging event up to every lunge of a dive, by comparing the rate of energy intake (*Ec*) with the total rate of energy intake (*En*) during each lunge, for dives with at least two lunge events.

### Statistics

The statistical analyses were performed with R2.15.2. (R Core team 2015). In order to confirm that post-surface duration is more associated to foraging dive duration than pre-surface duration, a generalized linear model (GLM) with gamma distribution was used. The response variable was foraging dive duration and the explanatory variables were pre-surface duration or post-surface duration. The Akaike Information Criteria (AIC) were compared between the two combination and the result with the smaller value was considered as the most parsimonious model. Correlations between patch residence time and the number of lunges per dive, as well as correlations between post-surface duration and dive duration were calculated as Spearman’s rank correlation coefficients. Statistical significance was set at P < 0.05. *Ec* of single lunge dives and *Ec* of the first lunge of multiple lunge dives were also compared using Man-Whitney *U* test. Statistical significance was set at P < 0.05.

Liner regression model was used to assess the relationship between *Ec* and *En* of each lunge number in order to visually observe the efficiency of each lunge event in a dive. According to the CPF, animals are expected to stop feeding when *En* is maximized. This is when *Ec* and *En* overlaps in the CPF model (Fig 1) and when *Ec* = *En* (*Ec*/*En* = 1). When the value of *Ec*/*En* > 1, *Ec* is still greater than *En*, thus the whales are expected to continue feeding. When *Ec*/*En* < 1, this implies that *Ec* has dropped below the point which maximum rate of energy intake can be obtained in that dive cycle, thus the whales are expected to stop feeding.

A statistical model was constructed to examine whether the presence/absence of other individuals influenced the patch residence time. The response variables were number of lunges per dive (*M*) and patch residence time (*T*); the explanatory variables were presence/absence of other individuals (*O*), dive depth (*D*), and maximum IPD (*MIPD*), and individual variations were set as the random effect. The lunge number was modeled using Poisson distribution,

M∼Poissson(λ) (3)

where λ is a mean value represented as,

$$\lambda =\exp(a+a_{O}O+a_{D}D+a_{MIPD}MIPD+r_{M,i})$$ (4)

where *a*_*O*_, *a*_*D*_ and *a*_*MIPD*_ are coefficients of presence/absence of other individuals (*O*), depth (*D*) and maximum IPD (*MIPD*), respectively. *O* takes 0 when other individuals were absent and 1 when other individuals were present. The *r*_*M*,*i*_ represents random effect of individuals on number of lunges per dive, where *i* represents the index of individuals. The patch residence time was modeled using Gamma distribution as

T∼Gamma(α,α/μ) (5)

where *α* is the shape parameter and *μ* is the mean value modeled as

$$\mu =M\exp(b+b_{O}O+b_{D}D+b_{MIPD}MIPD+r_{T,i})+(M-1)q.$$ (6)

The *b*_*O*_, *b*_*D*_ and *b*_*MIPD*_ are coefficients and *r*_T,i_ is the random effects of individual variation on duration per lunge. exp(*b*+*b*_*o*_*O*+*b*_*D*_*D*+*b*_*MIPD*_*MIPD*+*r*_*T*,*i*_) corresponds to the duration per lunge (Fig 1A; each red line). *q* represents the time interval between successive lunges. When there are *M* lunges in a dive, (*M-1*) intervals are included (for example, there are 3 intervals in 4 lunges dives as in Fig 1A). Hence, total patch residence time in a dive is modeled as above. *r*_*T*, *i*_ and *r*_*M*, *i*_ were assumed to be normally distributed.

$$r_{T,i}∼Normal(0,\sigma_{T}),r_{M,i}∼Normal(0,\sigma_{M}).$$ (7)

The prior distribution of *σ*_*T*_ and *σ*_*M*_ were set to be normal distributions whose means are 0 and standard deviations are 10. By conducting MCMC, posterior distributions and 95% confidence intervals (95% CI) for each parameter were computed. For MCMC sampling, the RStan library (rstan) was used. Among the parameters, the 95% CI of presence and absence of other animals (*a*_*O*_, *b*_*O*_), depth (*a*_*D*_, *b*_*D*_), and maximum IPD (*a*_*MIPD*_, *b*_*MIPD*_), were of special interest in this study.

### Ethics

The research was conducted with a research permit from the Icelandic Fishery Ministry (no permit number) and according to the Icelandic legislation and laws. No animal ethics approvals were required for this project in Iceland according to the Icelandic legislation and laws, but the experiments were conducted according to the guidelines of PLOS ONE for the treatment of animals in behavioral research.

## Results

### Foraging dive characteristics

**Fig 3:**
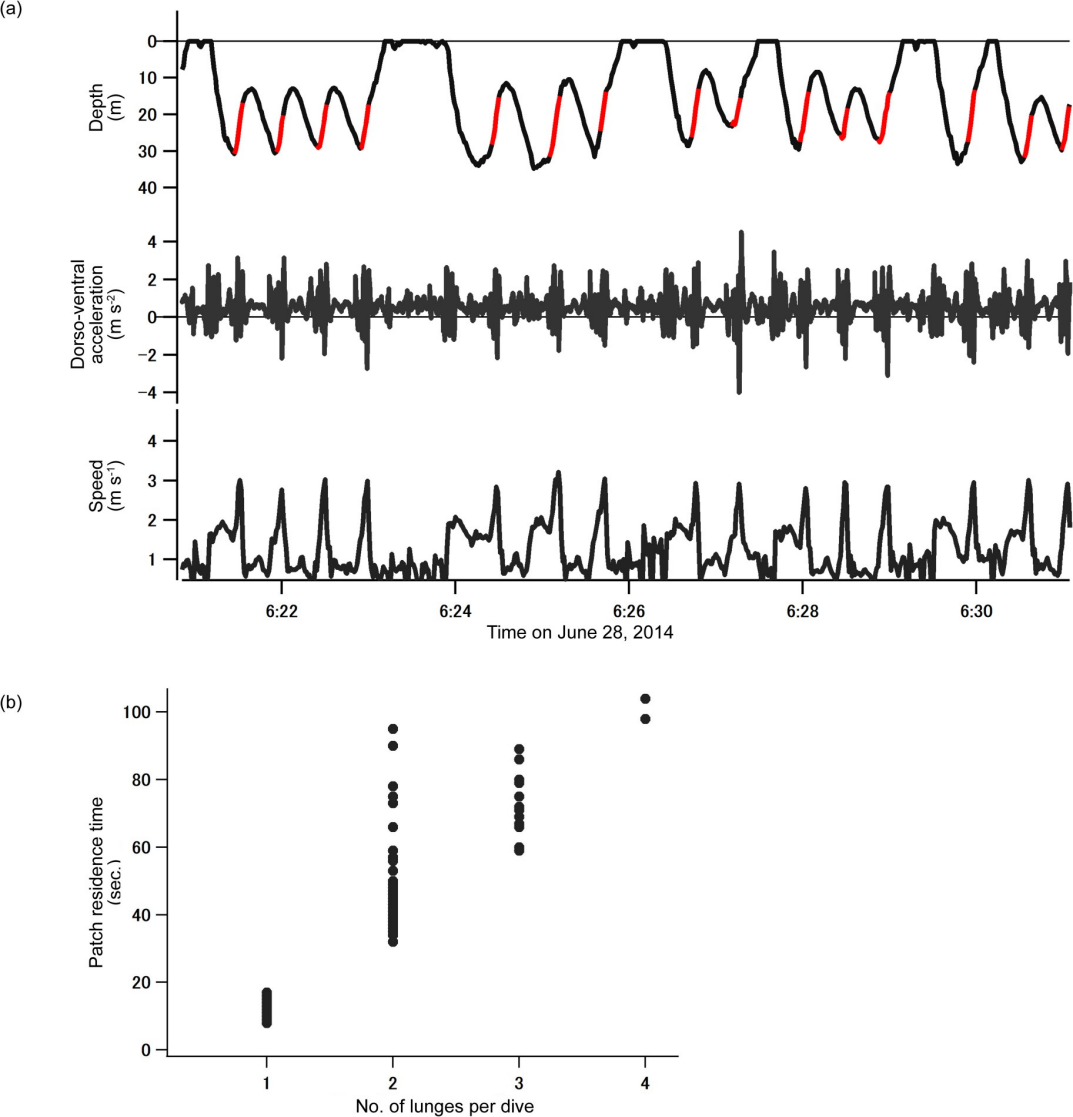
Characteristics of lunge dives. (a) Diving behavior of a humpback whale (ID: WhC14). Dive profile with lunge events (indicated in red), dorso-ventral acceleration, and swim speed in dives with varying numbers of lunges per dive (b) Relationship between patch residence time and number of lunges per dive, showing positive correlation (Spearman’s *ρ* = 0.78, P < 0.001).

Seven humpback whales were tagged in Skjálfandi Bay off Húsavík in northern Iceland. A total of 82 hours of behavioral data were obtained from these whales, as well as 45 hours of video data from six of them (Table 1). At our study site, humpback whales did not feed cooperatively; rather, they fed individually with vertical lunge feeding, identified from visual observation in field as well as from the peaks of simultaneous body acceleration during the ascent phase of dives (Fig 3A). There were a total of 2860 dives during the 82 hours and 1914 of them were accompanied by single lunge, or multiple lunges with a maximum of seven lunges per dive. The mean surface duration, dive duration, and dive depth of 2860 dives were 52.7 ± 115.6 sec, 85 ± 69.3 sec, and 27.8 ± 14.7 m, respectively (Table 1). The post-surface duration was more associated to foraging dive duration of humpback whales as first predicted. The AIC values for pre-surface model and post-surface model was, 19489.40 and 19232.19, respectively (n = 1991). The AIC value of post-surface duration was smaller thus, post-surface duration was used for constructing the foraging model.

**Table 1:** Tagging results of seven humpback whales.

ID	Date of attachment	Accelerometer (h)	Video (h)	Number of dives	Surface duration (sec.)	Dive duration (sec.)	Dive depth (m)
WhA13	5 Jun. 2013	4.1	N	218	15.9 ± 13.2	53.0 ± 39.4	19.3 ± 4.5
WhB13*	7 Jun. 2013	24.5	2.9	1315	28.5 ± 60.2	39.6 ± 46.8	17.0 ± 7.6
WhA14	25 Jun. 2014	12.5	11.5	216	125.0 ± 324.8	81.4 ± 109.6	21.5 ± 23.4
WhB14*	27 Jun. 2014	13.1	7.3	412	41.5 ± 114.6	73.6 ± 52.0	23.1 ± 10.1
WhC14*	28 Jun. 2014	17.2	12.5	493	30.9 ± 40.6	92.8 ± 100.4	29.9 ± 21.8
WhD14	29 Jun. 2014	6.6	6.6	132	67.6 ± 202.1	111.3 ± 66.0	36.7 ± 17.8
WhE14*	29 Jun. 2014	4.2	4.2	74	59.6 ± 53.8	143.0 ± 71.1	47.1 ± 17.5

The table shows whale ID, tagging date, and number of hours of accelerometer and video data obtained. Asterisks (*) indicate data used in the analyses. The general dive characteristics of all dives (feeding and non-feeding dives) from each whale are presented as mean value ± SD.

Krill was the only prey observed with the video-logger and relative density of prey in front of each whale was estimated from the video recordings throughout the dives. The feeding dive depth, indicated from depth profiles of each whale, ranged from 4 to 97 m and the mean was from 16 to 35 m. However, only data from dives shallower than 35 m were used for further analysis, as the images became too dark to make estimates at greater depths. Because all the feeding dives of two whales (ID: WhA14, WhD14) exceeded 35 m, the analysis was restricted to four whales (ID: WhB13, WhB14, WhC14, WhE14; Table 1) that performed 578 dives in total, including 251 dives with lunge events. The number of lunges counted in each foraging dive varied among dives, ranging from one to four with a median of one, and there was a positive correlation between patch residence time and the number of lunges per dive (Spearman’s *ρ* = 0.78, P < 0.001; Fig 3B).

### Foraging efficiency

**Fig 4:**
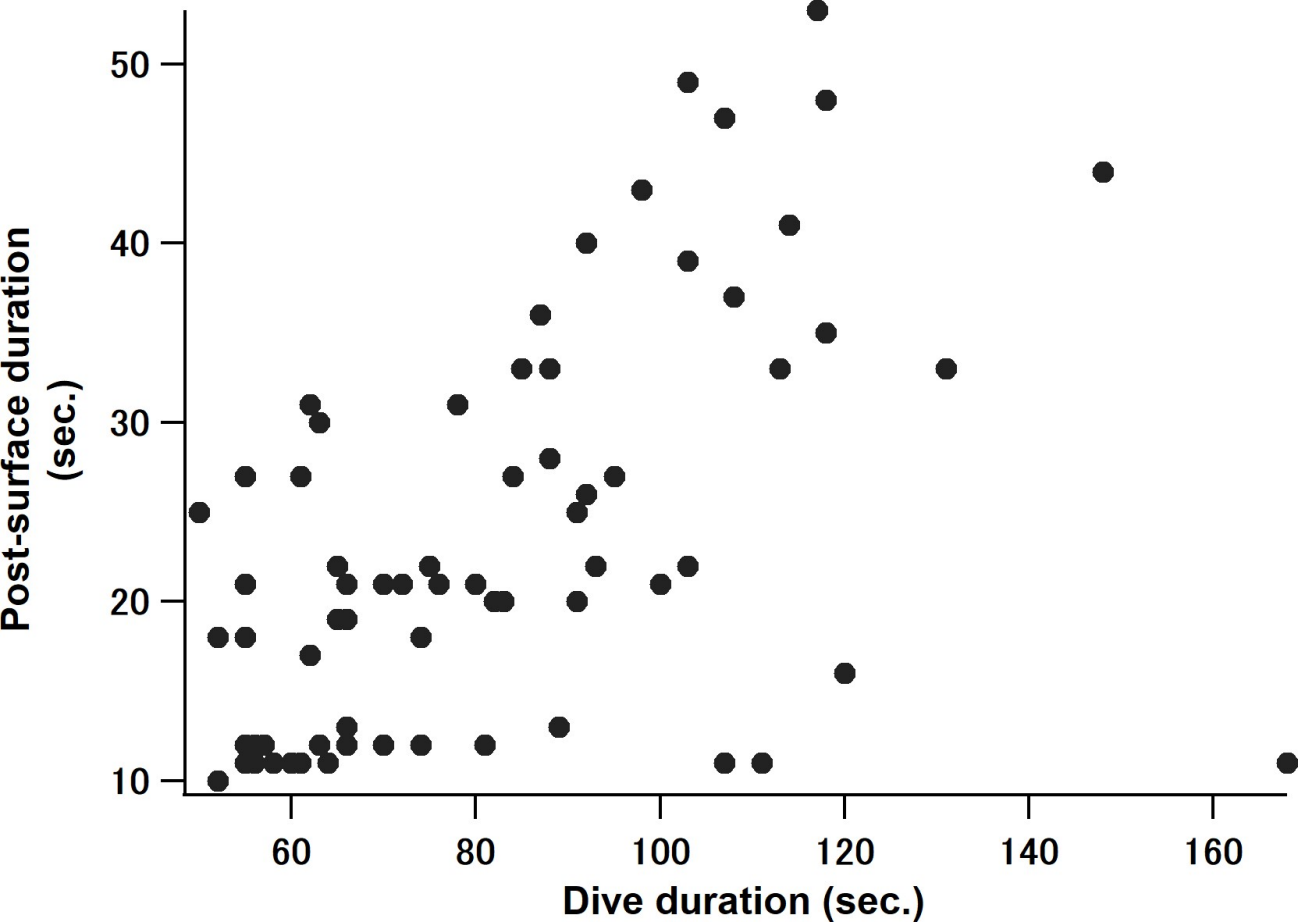
Relationship between dive duration and post-surface duration of foraging dives with greater than or equaled to two lunges within 35 m in depth. This showed positive relationship (Spearman’s *ρ* = 0.54, P < 0.0001; n = 70).

**Fig 5:**
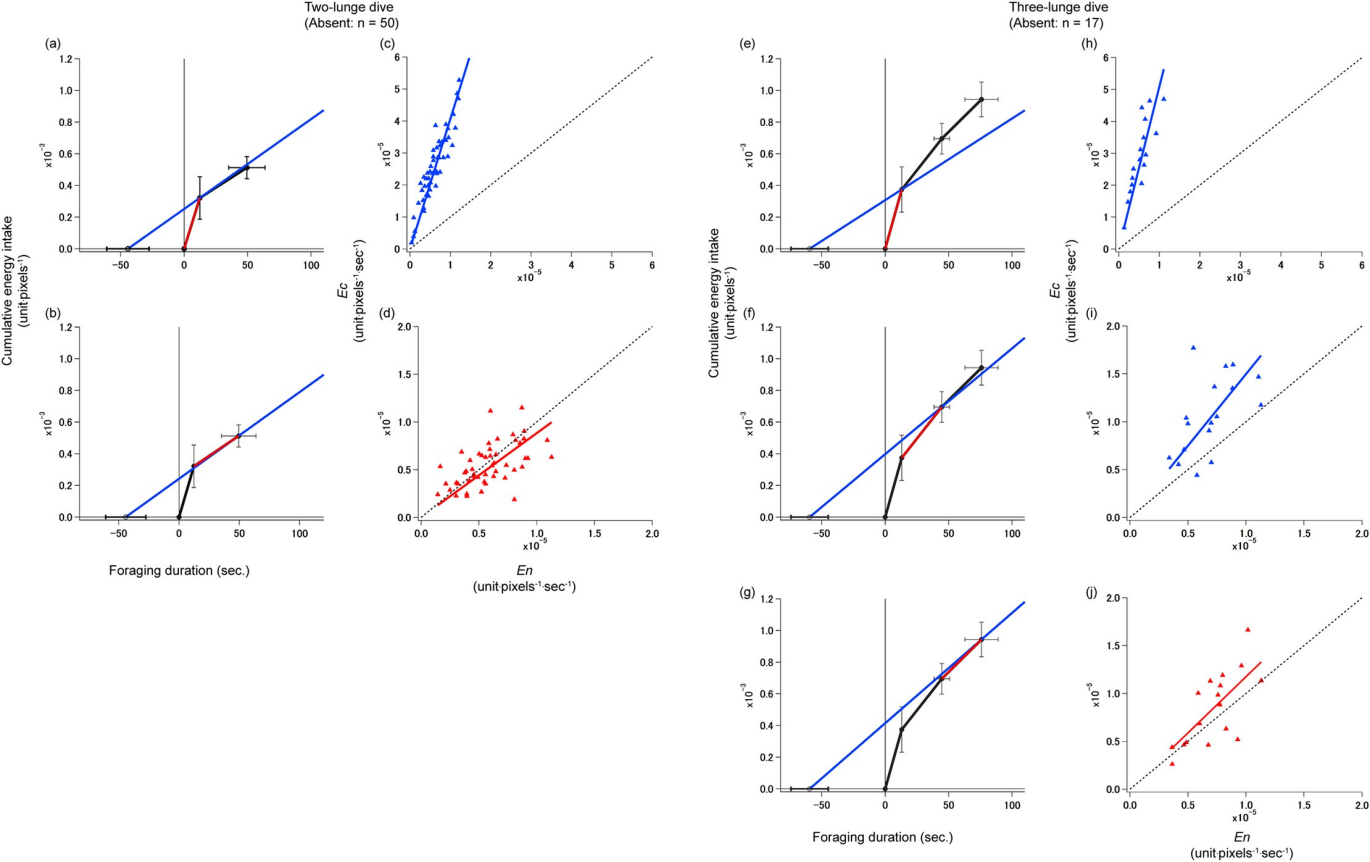
Foraging efficiency of lunges. Foraging models comparing the total rates of energy intake (*En*) and gain functions using mean values of dives with (ab) two-lunges (n = 50), (efg) three-lunges (n = 17). X-axis shows the foraging duration, composed of transit (descent + ascent) + post-surface + patch residence time. Y-axis shows cumulative energy intake. The total rate of energy intake (*En*) up to each lunge is represented by a blue line, and the rate of energy intake at each lunge (*Ec*) is indicated by a black or red line. Error bars on the black dots and the starting point of the blue line (*En*) represent the standard deviation of the mean values. A linear regression model of *Ec* versus *En* (cdhij) of the corresponding foraging model are shown on the right side of each foraging model. The dashed black line represents where *Ec/En =* 1. (c), (h), and (i) had *Ec*/*En* values greater than one (a; *Ec*/*En* = 4.1, 95% CI; 3.8–4.3: h; *Ec*/*En* = 5.1, 95% CI; 4.5–5.7: i; 1.5, 95% CI; 1.2–1.8). Although (j) also had *Ec*/*En* values greater than one the 95CI included *Ec/En =* 1 (*Ec*/*En* = 1.2, 95% CI; 0.99–1.3). (d) had *Ec*/*En* values less than one (*Ec*/*En* = 0.88, 95% CI; 0.75–0.98).

There were 70 feeding dives with at least two lunges, within the 251 feeding dives. To verify the consistency of our data with previous reports, the relationship between dive duration and post-surface duration was confirmed for these 70 feeding dives. This showed a positive relationship between post-surface duration and dive duration (Spearman’s *ρ* = 0.54, P < 0.0001; Fig 4), which agreed with the previous studies [16, 17]. Then, gain function of the foraging model was calculated for these 70 dives: 94% showed a decrease in rate of energy intake on the second lunge in a dive, and 90% showed a gradual decrease over lunges. The model for dives with two lunges indicated higher rates of energy intake (*Ec*) in comparison with the total rate of energy intake (*En*) during the first lunge (blue and red lines did not overlap in Fig 5A) having *Ec*/*En* equaled to 4.1 (95% CI; 3.8–4.3, Table 2: Fig 5C) However, during the last lunge in the CPF model (Fig 5B), the rates of energy intake (*Ec*) overlapped with the total rate of energy intake (*En*), having *Ec*/*En* equaled to 0.88 (95% CI; 0.79–0.98: Fig 5D). Statistically, this value is less than one, yet is a value very close to one. Dives with three lunges had higher *Ec* in comparison with *En* during the first (Fig 5E) and second lunges (Fig 5F); *Ec*/*En* of 5.1 (95% CI; 4.5–5.7) and 1.5 (95% CI; 1.2–1.8), respectively (Fig 5H and 5I). However, during the last lunge, again, *Ec* overlapped with *En* (Fig 5G), having *Ec*/*En* equaled to 1.2 (95% CI; 0.99–1.3: Fig 5J). Overall, the value of *Ec*/*En* of the three-lunge dive was greater than the *Ec*/*En* of the two-lunge dive. Statistical analysis could not be conducted for the case of dives with four lunges due to small sample sizes (n = 2); however, the dive had a similar trend with other dives (S1A–S1D Fig), with *Ec* and *En* overlapping on the last lunge (S1D Fig).

**Table 2:** Values of *Ec*/*En* and 95% CI for each lunge in a dive.

	*Ec* / *En*	95% CI
First lunge of two-lunge dive	4.1	3.8–4.3
Second lunge of two-lunge dive	0.88	0.79–0.98
First lunge of three-lunge dive	5.1	4.5–5.7
Second lunge of three-lunge dive	1.5	1.2–1.8
Third lunge of three-lunge dive	1.2	0.99–1.3

**Fig 6:**
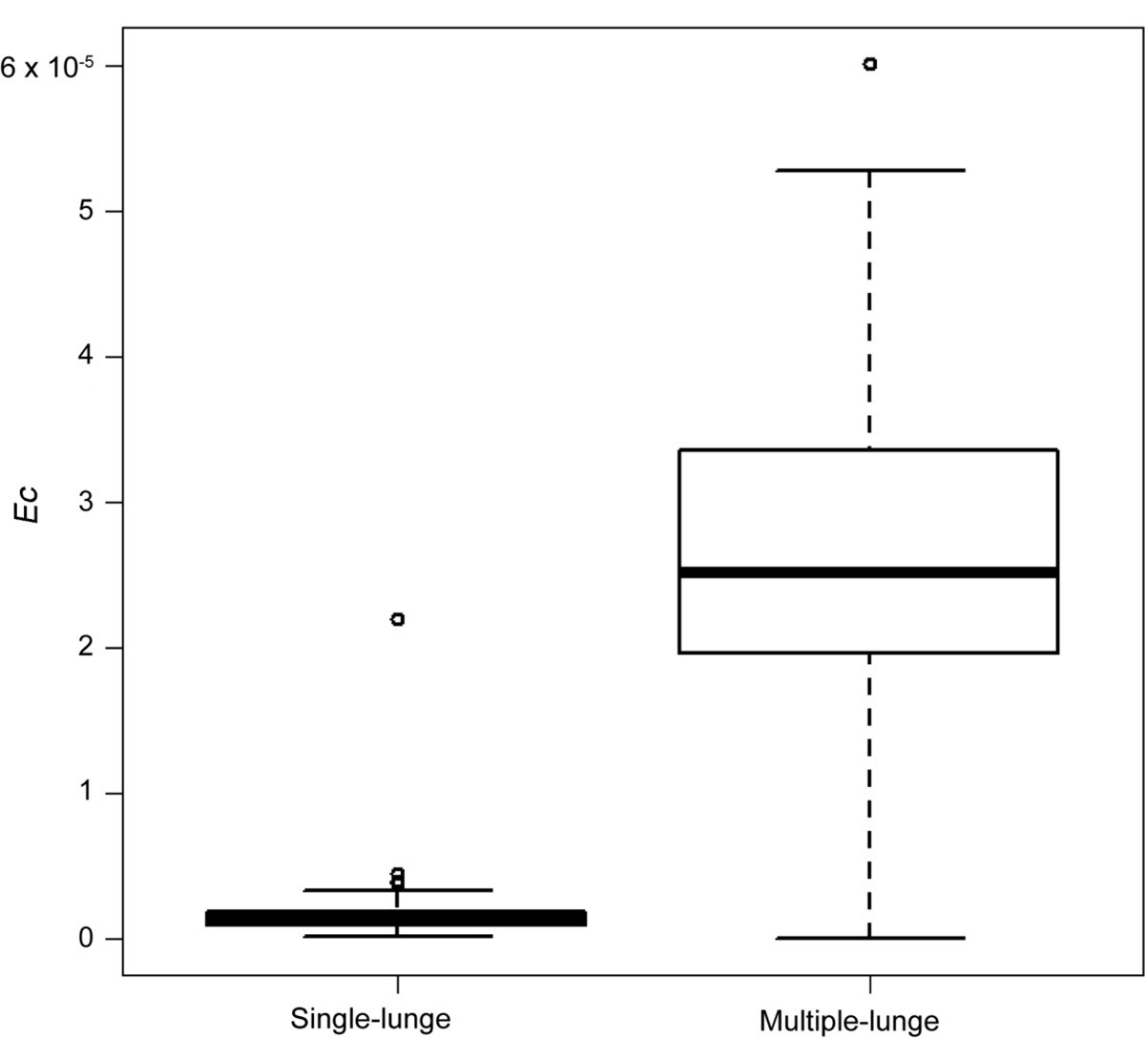
Comparison of *Ec* values of single lunge dives and first lunge of multiple lunge dives. The value of *Ec* for multiple lunge dives are significantly higher than that of single lunge dives.

Because foraging model could not be used to assess the single lunge dives (n = 155), we simply compared the *Ec* of the single lunge dives with *Ec* of the first lunge of multiple lunge dives. This showed a significant difference with higher *Ec* for multiple lunge dives (Mann-Whitney *U* test, P < 0.0001; Fig 6)

### Effect of other animals

**Fig 7:**
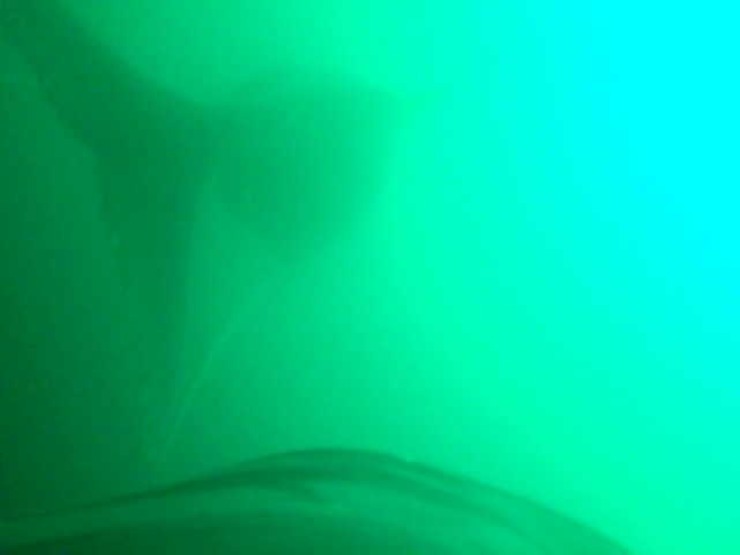
Snap shot from the video logger attached to a humpback whale. Another humpback whale is swimming in front of the tagged whale.

Other humpback whales were seen in 16 dives from two whales (Fig 7; ID: WhB14 and WhC14) and 12 out of those 16 dives were feeding dives. The maximum number of lunges in dives with no other animal was four, whereas it was two in dives with other individuals, which only occurred once, and all other foraging dives were single lunge dives. The gain function of this one dive with other humpback whale showed a noticeably lower values of the cumulative energy intake from the first lunge, producing shallower slopes overall then the dives with no other animals (Fig 5A and 5E, S1E and S1F Fig).

**Fig 8:**
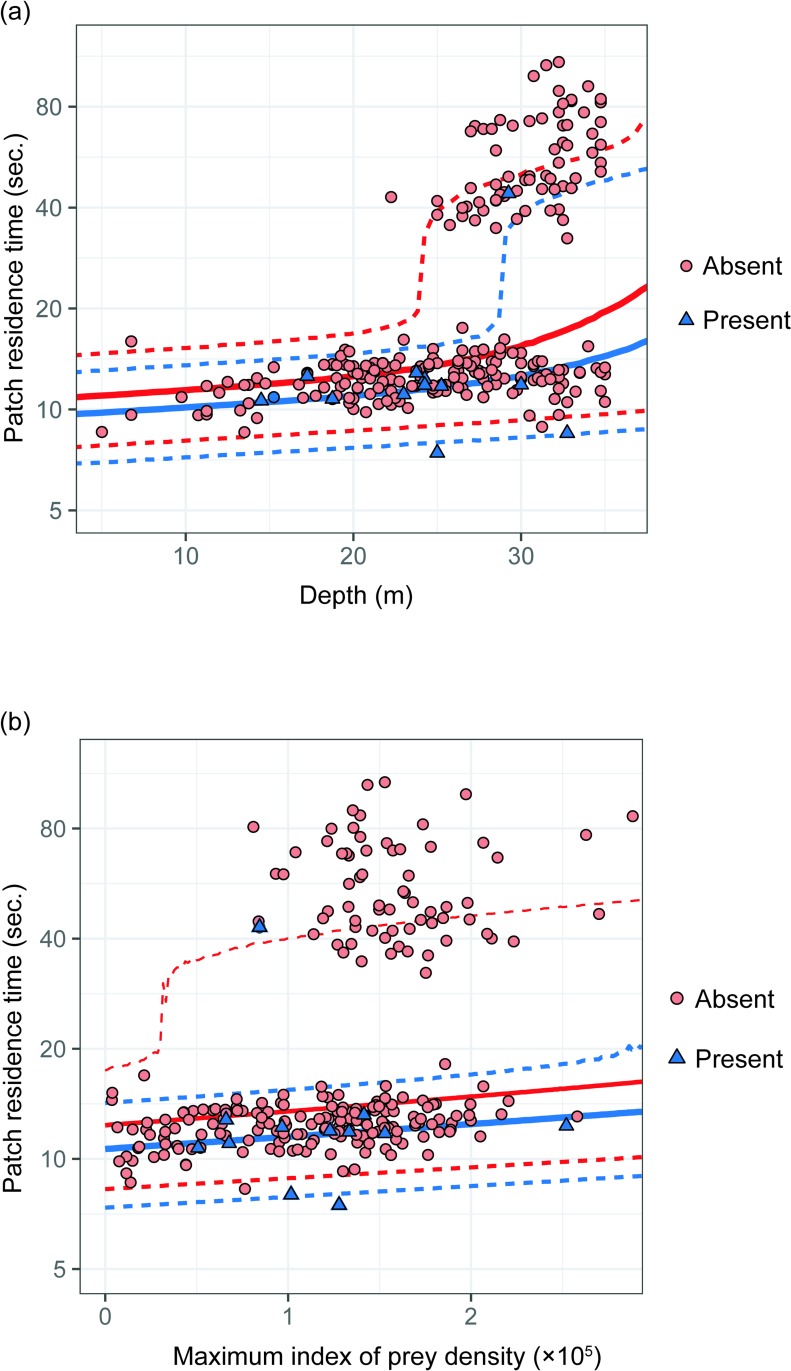
**Relationships between patch residence time and (a) dive depth and (b) the maximum Index of Prey Density (IPD) for four whales.** The red points represent absent (n = 232) dives and blue triangles represent present dives (n = 12). The points show the patch residence time, rescaled so that the effect of MIPD is normalized to its mean value in (a), and the effect of depth is normalized to its mean value in (b). The solid lines represent the mean values and the dashed lines represent the 95% prediction intervals of posterior distribution computed from the MCMC simulation. For computing 95% prediction intervals, MIPD was set to its mean value in (a) and depth was set to its mean value in (b).

Patch residence time in a single dive was shorter when humpback whales encountered other individuals (Fig 8). The negative value of *a*_*O*_ and *b*_*O*_ (Table 3) indicates that the presence of other individuals decreases the lunge number, and the duration per lunge. Based on the 95% CI of *a*_*O*_ and *b*_*O*_, the effect of presence/absence of other individuals was significant for duration per lunge (*b*_*O*_), but not for number of lunges per dive (*a*_*O*_). The values of *a*_*D*_, *b*_*D*_, *a*_*MIPD*_ and *b*_*MIPD*_ were all positive (Table 3). This indicates that the lunge number and duration per lunge increases as depth and maximum IPD increases; as a result, increase in patch residence time (Fig 8). These effects were all significant except for MIPD on number of lunges (*a*_*MIPD*_) (Table 3).

**Table 3:** 95% CI for each parameter based on prior distributions computed by MCMC sampling.

Coefficients (explanatory variable)	Mean	95% confidence intervals		
a_D_ (depth)	0.18	0.11	-	0.23
a_MIDP_ (MIDP)	0.31	- 0.19	-	0.71
a_O_ (others)	-1.1	-4.3	-	0.63
b_D_ (depth)	0.0068	0.0016	-	0.011
b_MIDP_ (MIDP)	0.068	0.011	-	0.11
b_O_ (others)	-0.12	-0.21	-	-0.031

## Discussion

Assessing the predator–prey interactions of free-ranging diving marine predators is challenging. When studying the predator–prey interactions of rorquals, ship-mounted echo-sounders were commonly used to measure the distribution and abundance of prey near tagged whales in previous studies [12, 17, 21, 22, 39–41]. The advantage of this method is that it maps the prey distribution in the feeding grounds of whales at wider and deeper ranges over a long period of time. Recent study of humpback whales using a ship-mounted echo-sounder revealed that the foraging decisions of humpback whales are driven by both prey depth and density. Humpback whales maximized the energy intake over time by mainly feeding at a shallow depth to minimize their diving and search cost and to increase overall feeding rate [22]. Their study provided new and interesting insights on the ecological decision-making of foraging humpback whales.

The disadvantage of their previous method was that it could not reveal the temporal changes in prey density during each feeding event; therefore, momentary information on the prey density encountered by whales in the time and places where feeding occurred was not obtained. Our new method filled this gap and assessed the relative prey density from the video loggers attached to the whales, providing information on the temporal changes in prey density in front of the whale and linked it to their behavior.

Our study indicated a statistically significant positive relationship between patch residence time (number of lunges per dive) and depth, which agreed with the previous report by Friedlaender et al. [23]. Patch residence time also increased with maximum IPD (Fig 8B), although this was not statistically significant (Table 3). This may be because our analysis was restricted to shallow depth, and krill patch density within 35 m was fairly constant. Yet, this method allowed us to validate the central assumption of optimal foraging theory (i.e. the diminishing return), and to test the hypothesis based on CPF (i.e. the energy intake rate maximization strategy within a single dive cycle). The rate of energy intake (*Ec*) at each lunge showed a gradual decrease through consecutive lunge events in the majority (90%) of dives (diminishing return). This may be an indication of decrease in prey density due to large amount of prey consumed during each lunge event, or prey being dispersed from passing through the aggregated patch of prey on the previous lunge as we also found decrease in mean IPD over consecutive lunge events in a single dive (S1 Table). Under the condition of diminishing return, humpback whales stopped feeding when the slopes of total rate of energy intake (*En*) and rate of energy intake *(Ec)* overlapped on the model (*Ec*/*En* was very close to one or less). This implies that humpback whales were efficiently feeding by adjusting their foraging duration or number of lunges per dive in relation to decreasing prey density so that the rate of energy intake in a single dive cycle is nearly maximized. Some studies of optimal foraging for air-breathing divers considered the physiological constraint of oxygen store and predicted how air-breathing divers should allocate time for foraging and surface duration to maximize the total rate of energy intake per dive [5, 10, 42]. In these studies, energy intake was assumed to be linear (i.e. no diminishing return, however, see [43, 44]) and the foraging duration to be strongly constrained by the diminishing cumulative oxygen uptake [13]. In this study, however, the gain function diminished drastically, and foraging duration was expected to be more constrained by diminishing rate of energy intake (ecological constraint) than the oxygen store (physiological constraint). Hence, we did not consider the oxygen store as a constraint for simplicity of this study.

One of the difficult issues for constructing our foraging model was, which post-surface duration to be used to calculate *En* for lunges before the last lunge. Theoretically, as our data indicated (Fig 4), less lunge and patch residence time may have less post-surface duration. However, there were still variations in post-surface duration even within dives with same lunge numbers and we never know how long the animals have stayed at surface if they stopped feeding earlier. Thus, to keep our model simple, and also because the significance of our study is to use actual data, we used the actual post-surface duration (mean post-surface duration of that lunge number in case of Fig 5) of that certain dive to calculate all *En* of that lunges. Perhaps, for our shallow and short dives with small number of lunges per dive, this was not a big issue affecting our results. However, in the future when we deal with longer and deeper foraging dives, this issue should be more carefully considered because post-surface duration may start to make a difference to the results.

Additionally, our video-based method provided us an unexpected opportunity to assess predator–prey–competitor interactions in humpback whales. Our analysis showed a decrease in patch residence time in the presence of other humpback whales (Fig 8). This suggests that not only depth and prey density, but also the presence/absence of other animals affects the foraging behavior of humpback whales. Other humpback whales in this field are recognized as direct competitors feeding on the same patch of krill. Humpback whales fed until the total rate of energy intake (*En*) in a single dive cycle was maximized when alone (Fig 5, S1A–S1D Fig). In comparison, when other humpback whales were present, the patch residence time was shorter and most dives only contained one. Thus, in presence of competitors, humpback whales might have left the patch or ended the dive early because patch quality was already decreased by the foraging activity of other individuals or considering the potential decrease in prey abundance due to competition (S1E and S1F Fig). Although sample size of dive with other individuals was rather small in this study, our results show an interesting trend and introduce a new potential to quantitatively investigate the effect of other individuals on feeding top predators in natural condition.

The limitation of our method using video cameras was the narrow field-of-view, with information on prey being restricted to close proximity in front of the whales, due to poor water clarity and limited light at greater depths. At shallow depths, the light condition was fairly constant at all times of the day, because our study was conducted during the season of midnight sun. The placement of the tag might also differ in each tagging attempt and over time, because the tags shift. As a result, it was not possible to estimate the prey density from the same angle-of-view. Still, in most cases, the tag shifted after attachment to be aligned with the water flow facing forward; thus, we assumed that the levels of krill flowing by the whales as they swam through the patch reflected the quality of the patch, regardless of where the camera was attached. The quality of our method is not enough to estimate the absolute prey density or precise consumption rate. However, the most important part of this study was not to estimate the actual energy intake, but the shape of the gain function. Stephens and Krebs [6] stated the importance of specifying the energy intake over time, as this information determines the shape of the gain function, otherwise, foraging models are meaningless. Our method could simultaneously obtain the levels (high or low density) of prey information in front of the whale and the time that the feeding occurred. The shape of gain functions plotted using the data from our method should provide a good reflection of the relative energy intake of the whales over their patch residence time, allowing us to investigate foraging dive efficiency. Thus, we believe that our approach is the most effective and the only way to complete such a study based on existing technology. This study can be further improved by combining the method using an echo-sounder, giving us the information of both short-term and long-term change in prey density and distribution over time. Moreover, there have been vast improvements in technology over the past couple of years and some researchers are starting to use 360° cameras. This could resolve many of the issues in this study, giving us better estimation of change in prey density and the observation of other animals around the tagged whale from all directions.

Optimal foraging theory is undoubtedly a powerful tool to investigate the foraging behavior of animals. Using the concept of CPF, we found that the humpback whales in Skjálfandi Bay are taking the rate maximizing strategy of energy intake in each foraging cycle. We have observed other humpback whales around the tagged whales which showed some trend of change in behavior. This emphasizes the importance of monitoring and incorporating as much information on the surrounding environment, to fully understand the foraging decision of wild animals. Our method may be an effective way to quantitatively investigate predator–prey–competitor interactions in the context of CPF for future studies.

## Supporting information

S1 Fig Foraging efficiency of four lunge dive and present dive. Foraging models comparing the total rates of energy intake (*En*) and gain functions using mean values of dives with (abcd) four-lunges when other animals are absent (n = 2); and (ef) two-lunges when other animals are present (n = 1). X-axis shows the foraging duration, composed of transit (descent + ascent) + post-surface + patch residence time. Y-axis shows cumulative energy intake. The total rate of energy intake (*En*) up to each lunge is represented by a blue line, and the rate of energy intake in each lunge (*Ec*) is indicated by a black or red line. Error bars on the black dots represent the standard deviation of the mean values.
(TIF)

S1 Table Mean IPD and mean duration of lunges. (PDF)

S1 File Data used for Fig 3B and Fig 8. The file includes data for statistical analysis regarding these figures.
(XLSX)

S2 File Data used for Fig 5 and S1 Fig. (XLSX)
