# Supplementary figures and images for: Leave or stay? Video-logger revealed foraging efficiency of humpback whales under temporal change in prey density
Source: PLoS One. 2019 Feb 5;14(2):e0211138. doi: 10.1371/journal.pone.0211138 (PMC6363283; doi:10.1371/journal.pone.0211138)

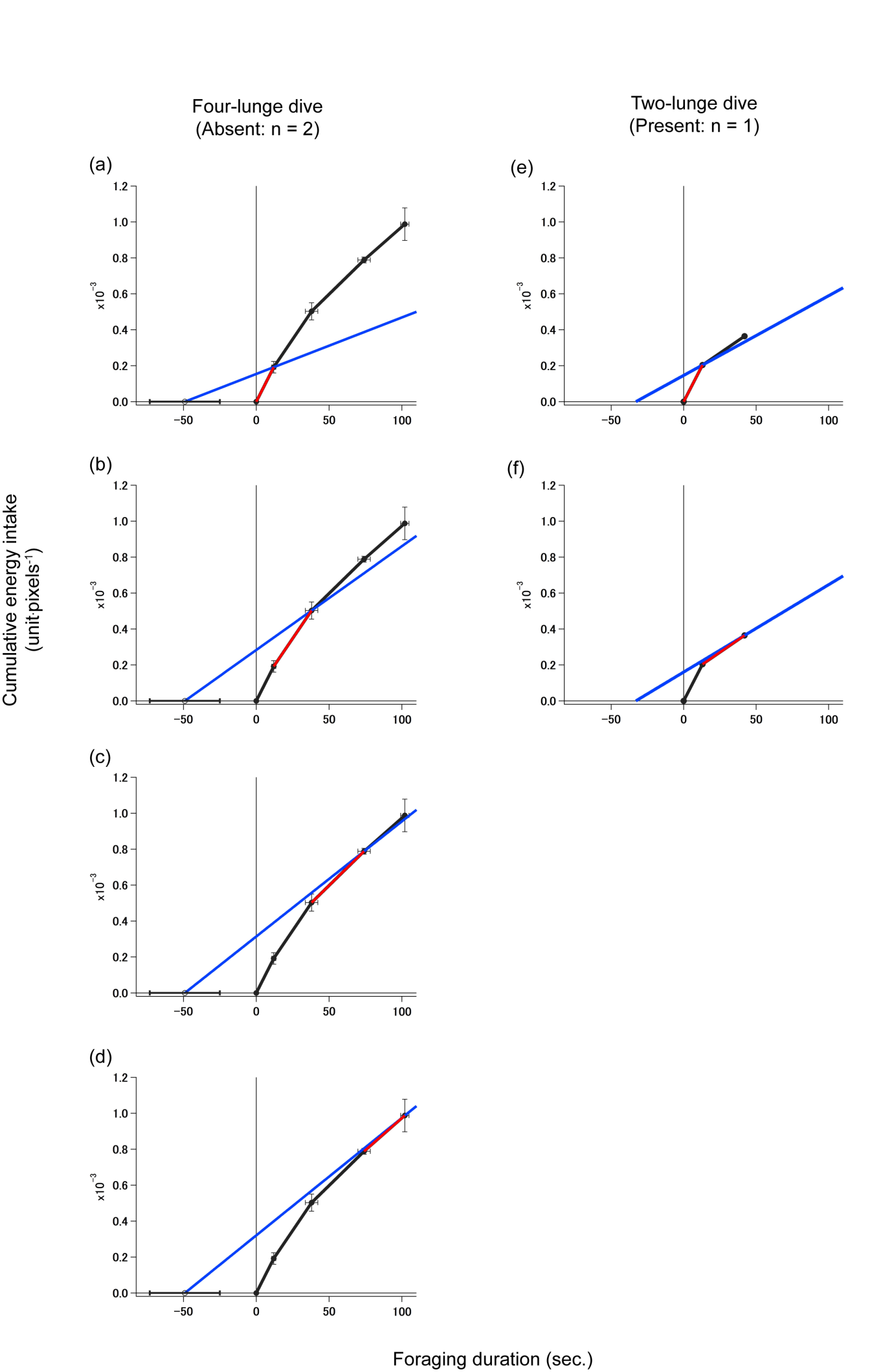

Supplement: S1 Fig — Foraging models comparing the total rates of energy intake (En) and gain functions using mean values of dives with (abcd) four-lunges when other animals are absent (n = 2); and (ef) two-lunges when other animals are present (n = 1). X-axis shows the foraging duration, composed of transit (descent + ascent) + post-surface + patch residence time. Y-axis shows cumulative energy intake. The total rate of energy intake (En) up to each lunge is represented by a blue line, and the rate of energy intake in each lunge (Ec) is indicated by a black or red line. Error bars on the black dots represent the standard deviation of the mean values. (TIF) [file pone.0211138.s001.tif]
