# Supplementary material for: Leave or stay? Video-logger revealed foraging efficiency of humpback whales under temporal change in prey density
Source: PLoS One. 2019 Feb 5;14(2):e0211138. doi: 10.1371/journal.pone.0211138 (PMC6363283; doi:10.1371/journal.pone.0211138)
Supplement: S1 Table — (PDF) [file pone.0211138.s002.pdf]

S1 Table. Mean IPD (s.d.) and mean duration of lunges (s.d.).

	1st lunge		2nd lunge		3rd lunge		4th lunge	
	Mean IPD	Duration (sec)	Mean IPD	Duration (sec)	Mean IPD	Duration (sec)	Mean IPD	Duration (sec)
Single lunge dives (n = 154)	1.4×10^{-5} (7.5×10^{-6})	12.6 (1.4)						
Two lunge dives (n = 50)	3.1×10^{-5} (6.5×10^{-6})	12 .4 (1.1)	1.5 x 10^{-5} (6.4×10^{-6})	12 .9 (1.2)				
Three lunge dives (n = 17)	3.0×10^{-5} (1.1×10^{-5})	13 .1 (0.8)	2.8×10^{-5} (9.9×10^{-6})	12.9 (1.0)	1.9 x 10^{-5} (7.6×10^{-6})	12.9 (1.3)		
Four lunge dives (n = 2)	2.1×10^{-5} (1.1×10^{-5})	12.0 (1.4)	2.7×10^{-5} (5.8×10^{-6})	11.5 (0.7)	2.1×10^{-5} (7.8×10^{-8})	13.5 (0.7)	1.7×10^{-5} (9.5×10^{-6})	12.0 (1.4)
